# Stage IIIC Bilateral Dysgerminoma in 46,XY Swyer Syndrome: Preventing Malignancy Through Early Endocrine Evaluation of Primary Amenorrhea

**DOI:** 10.1002/ccr3.73154

**Published:** 2026-07-12

**Authors:** Chukwuka Elendu, Alamjeet K. Sidhu, Olisa S. Okabekwa, Ayi T. Debua, Ahmed I. Ali

**Affiliations:** ^1^ Federal University Teaching Hospital Owerri Nigeria; ^2^ Connecticut Institute for Communities Danbury USA; ^3^ University of Nigeria Teaching Hospital Enugu Nigeria; ^4^ University of Calabar Teaching Hospital Calabar Nigeria; ^5^ Medical Academy Named After S.I. Georgievsky of Vernadsky Simferopol Russia

## Abstract

Primary amenorrhea requires systematic endocrine evaluation before empirical hormonal therapy. In adolescents with hypergonadotropic hypogonadism, prompt karyotype analysis is essential to identify 46,XY complete gonadal dysgenesis (Swyer syndrome), enabling timely prophylactic gonadectomy, preventing gonadal malignancy, and improving endocrine, reproductive, and long‐term clinical outcomes.

Dear Editor,

1

We read with great interest the recent report describing a 16‐year‐old phenotypic female with 46,XY complete gonadal dysgenesis (Swyer syndrome) who presented with primary amenorrhea and was ultimately diagnosed with FIGO Stage IIIC bilateral dysgerminoma following delayed endocrine evaluation [1].

Swyer syndrome is a rare form of complete gonadal dysgenesis, with an estimated incidence of approximately 1 in 80,000 live births, characterized by a 46,XY karyotype, failure of testicular differentiation during embryogenesis, hypergonadotropic hypogonadism, streak gonads, and persistence of Müllerian structures despite a phenotypically female appearance [2, 3]. Although pathogenic variants in SRY, MAP3K1, NR5A1, DHH, SOX9, and other genes have been implicated, nearly half of affected individuals lack an identifiable molecular diagnosis, reflecting the disorder's marked genetic heterogeneity [2, 3, 4]. Irrespective of the underlying molecular defect, dysgenetic gonads containing Y‐chromosomal material carry a 30%–45% lifetime risk of gonadoblastoma and dysgerminoma, increasing with age, making early diagnosis and prophylactic bilateral gonadectomy essential for cancer prevention (Figure 1) [3, 4, 5, 6].

**FIGURE 1:**
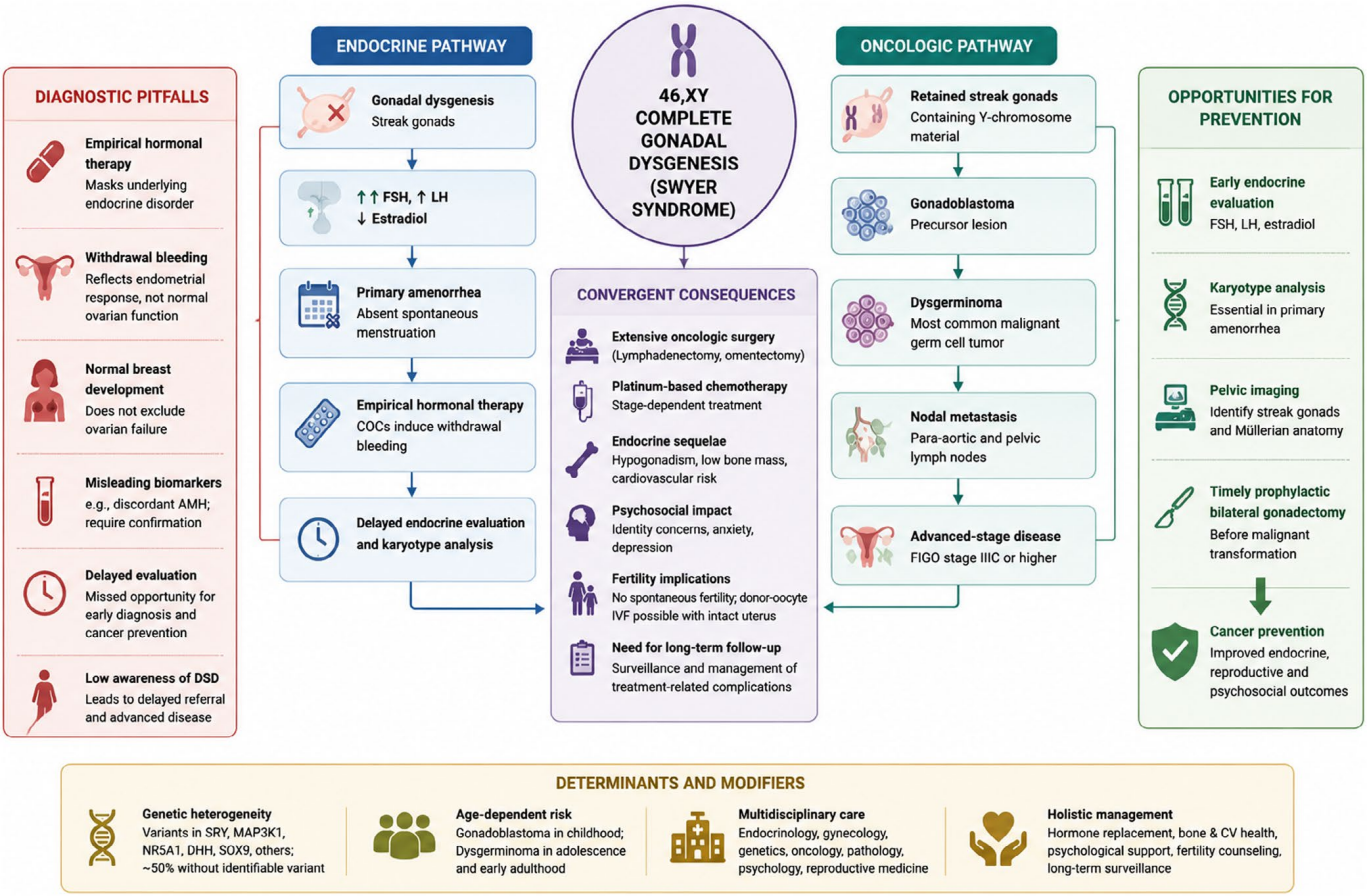
Integrated endocrine‐oncologic pathway highlighting diagnostic pitfalls and opportunities for prevention in Swyer syndrome.

Particularly striking in the report is the prolonged interval between the patient's initial menstrual abnormality and definitive endocrine evaluation. Combined oral contraceptives induced withdrawal bleeding despite the persistent absence of spontaneous menstruation, creating the appearance of preserved menstrual function while the underlying endocrine disorder remained undiagnosed [1]. Because withdrawal bleeding following exogenous estrogenprogestin therapy reflects only endometrial responsiveness and does not establish normal hypothalamic‐pituitary‐gonadal function, empirical hormonal treatment before a definitive evaluation may delay the diagnosis of serious endocrine disorders, including disorders of sex development, premature ovarian insufficiency, and pituitary disease [7, 8]. Current endocrine and gynecologic guidelines therefore recommend systematic evaluation with gonadotropins, estradiol measurement, pelvic imaging, and karyotype analysis in adolescents with hypergonadotropic hypogonadism or absent pubertal progression [7, 8, 9].

The endocrine profile provided compelling evidence of gonadal failure before malignancy was recognized, with markedly elevated follicle‐stimulating hormone and luteinizing hormone concentrations accompanied by profoundly suppressed estradiol, findings characteristic of primary gonadal insufficiency rather than hypothalamic or pituitary dysfunction [1]. In this setting, karyotype analysis should be an integral component of the initial diagnostic evaluation, as failure to identify Y‐chromosomal material may leave dysgenetic gonads at continued risk of malignant transformation [3, 5, 7].

Another notable finding is the preservation of apparently normal secondary sexual characteristics despite complete gonadal dysgenesis, a phenotype attributable to peripheral aromatization of adrenal androgens or transient exogenous estrogen exposure, as occurred during oral contraceptive therapy in this patient [1, 2, 6]. Accordingly, Tanner stage and breast development should not be regarded as reliable indicators of ovarian function but interpreted alongside biochemical assessment, particularly in adolescents presenting with primary amenorrhea.

The discrepancy between the initial anti‐Müllerian hormone (AMH) concentration and the subsequently undetectable repeat measurement highlights the importance of interpreting endocrine biomarkers within their clinical context [1]. Because AMH is secreted by granulosa cells of growing ovarian follicles, detectable concentrations are unexpected in complete gonadal dysgenesis. Failure to reproduce the initial result suggests analytical variability or assay interference, emphasizing the need to confirm discordant findings before clinical interpretation, particularly when laboratory results conflict with the overall clinical picture [10]. Accordingly, isolated biochemical abnormalities should not outweigh consistent clinical, cytogenetic, and radiologic evidence.

Bilateral dysgerminoma with nodal metastasis underscores the malignant potential of retained dysgenetic gonads (Figure 2) [1]. Dysgerminoma, the most common malignant germ‐cell tumor in Swyer syndrome, frequently arises from pre‐existing gonadoblastoma, although de novo transformation has also been described [5, 6]. Despite excellent responsiveness to platinum‐based chemotherapy, prognosis remains stage dependent [11, 12]. These findings further support early prophylactic gonadectomy following the diagnosis of 46,XY complete gonadal dysgenesis as the most effective strategy for preventing malignant transformation [3, 5, 6].

**FIGURE 2:**
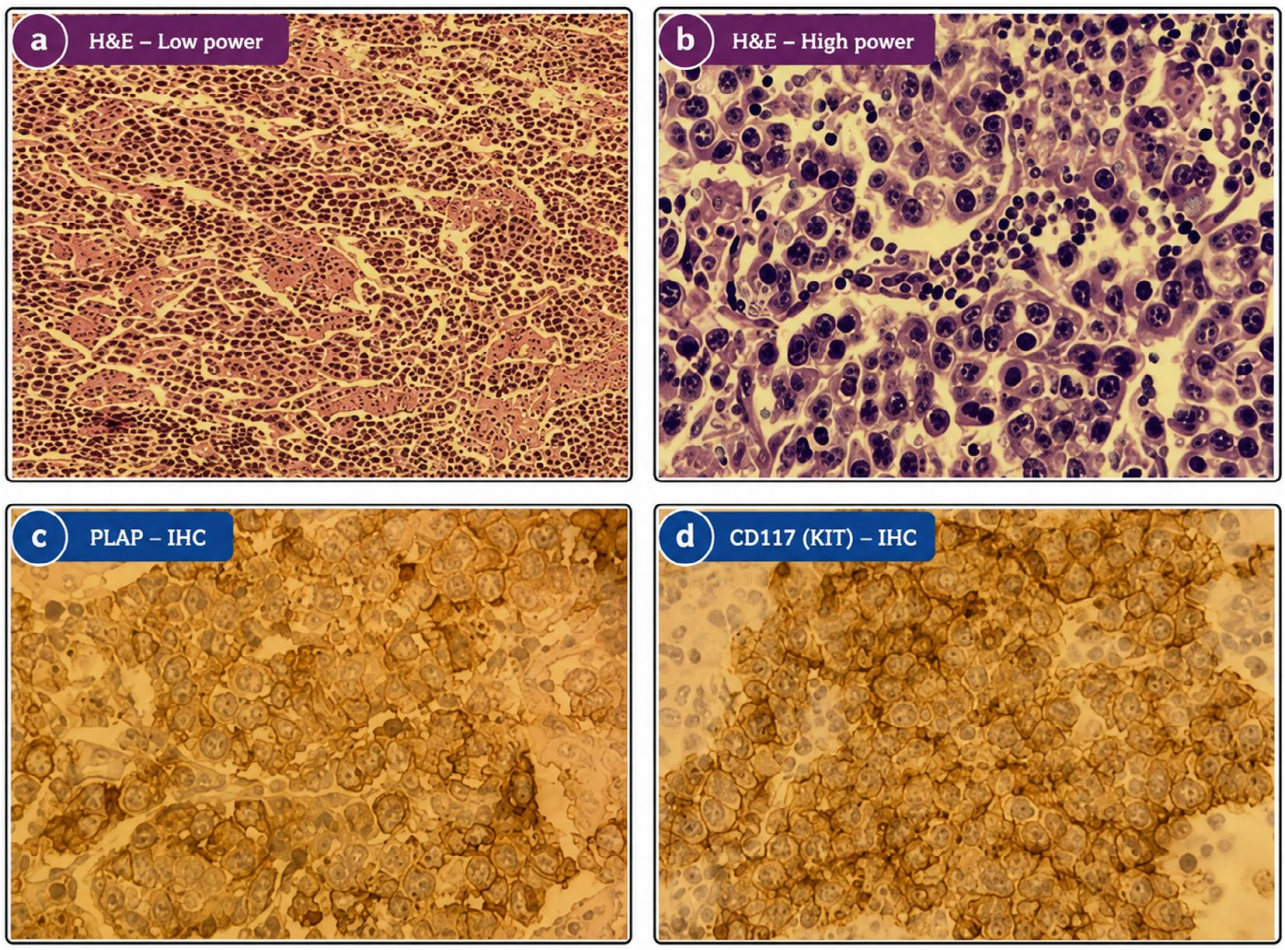
Histopathological and immunophenotypic features of ovarian dysgerminoma showing characteristic H&E morphology (a,b) and positive PLAP (c) and CD117 (KIT) (d) immunohistochemical staining. Reproduced from [1] under the CC BY License.

Beyond tumor prevention, Swyer syndrome requires multidisciplinary management involving pediatric endocrinologists, gynecologists, clinical geneticists, reproductive specialists, pathologists, and psychologists. Care should encompass hormone replacement therapy, optimization of bone mineral accrual and cardiovascular health, fertility counseling, psychosocial support, and long‐term surveillance for treatment‐related complications, particularly during adolescence when delayed diagnosis may have profound psychological as well as oncologic consequences [2, 7, 9].

The advanced stage at presentation also emphasizes the limitations of relying solely on symptoms to guide the evaluation of adolescents with primary amenorrhea. The absence of abdominal pain, constitutional symptoms, or palpable pelvic masses does not exclude gonadal malignancy, as abdominal and pelvic examinations remained unremarkable despite bilateral ovarian tumors, a para‐aortic metastatic mass, and lymph node involvement [1]. These findings support a low threshold for pelvic ultrasonography and magnetic resonance imaging when endocrine features suggest gonadal dysgenesis, as imaging not only delineates Müllerian anatomy and identifies streak gonads but also facilitates early detection of malignant transformation before dissemination [7, 8].

The tumor marker profile further underscores the value of biochemical assessment in suspected germ cell tumors. Markedly elevated lactate dehydrogenase (LDH), together with normal alpha‐fetoprotein (AFP), borderline β‐human chorionic gonadotropin (β‐hCG), and normal CA‐125 levels, is characteristic of dysgerminoma and emphasizes the importance of interpreting tumor markers collectively [1, 11]. Although LDH lacks specificity, it remains a useful biomarker of dysgerminoma, whereas normal AFP helps exclude yolk sac tumor and markedly elevated β‐hCG suggests syncytiotrophoblastic differentiation or alternative germ cell histologies [11, 12, 13]. Combined with endocrine evaluation, this biochemical profile improves preoperative diagnostic accuracy and guides definitive surgical management [11, 12, 13].

International guidelines recommend bilateral gonadectomy soon after the diagnosis of complete 46,XY gonadal dysgenesis because dysgenetic gonads carry an age‐dependent risk of malignant transformation, with precursor lesions such as gonadoblastoma detectable in childhood and progression to invasive dysgerminoma during adolescence or early adulthood [3, 4, 5, 6]. Delaying surgery until symptoms develop forfeits an opportunity for cancer prevention, as metastatic disease may necessitate extensive oncologic surgery, including lymphadenectomy, omentectomy, and adjuvant chemotherapy, rather than prophylactic gonadectomy [1].

Molecular diagnostics have identified pathogenic variants in an increasing proportion of individuals with 46,XY disorders of sex development; however, negative genetic testing should not delay clinical management once cytogenetic and endocrine findings establish complete gonadal dysgenesis [2, 3, 4]. Beyond establishing the molecular etiology, genetic testing improves understanding of disease mechanisms, facilitates genetic counseling, and informs recurrence risk, but the risk of gonadal malignancy is determined primarily by the presence of Y chromosome–containing dysgenetic gonads rather than by the specific genetic defect. Prophylactic gonadectomy therefore remains indicated irrespective of whether a pathogenic variant is identified [3, 4, 5, 6].

Because adolescence is a critical period for psychosexual development and identity formation, disclosure of an unexpected 46,XY karyotype following the diagnosis of advanced malignancy may impose substantial emotional distress. These challenges underscore the importance of longitudinal counseling addressing hormone replacement, bone and cardiovascular health, fertility options, and psychosocial adaptation [7, 8, 9, 10]. Although spontaneous fertility is not possible in complete gonadal dysgenesis because of the absence of functional ovarian tissue, pregnancy through donor‐oocyte in vitro fertilization remains feasible in appropriately prepared individuals with an intact uterus, highlighting the importance of early diagnosis for reproductive planning [2, 8].

Despite well‐established diagnostic algorithms, empirical hormonal therapy may still precede definitive evaluation of primary amenorrhea, masking symptoms, delaying referral, and overlooking clinically significant conditions such as Swyer syndrome. Hypergonadotropic hypogonadism in adolescents should prompt immediate karyotype analysis regardless of phenotypic appearance, and apparent breast development should not be interpreted as evidence of preserved ovarian function when biochemical findings indicate gonadal failure. Greater adherence to these principles across primary care, adolescent medicine, gynecology, and endocrinology could reduce diagnostic delays and prevent avoidable malignant transformation [7, 8, 9].

Greater awareness of disorders of sex development among clinicians evaluating primary amenorrhea is essential to promote an etiologic diagnosis rather than empirical treatment. Standardized diagnostic pathways incorporating hormonal evaluation, pelvic imaging, and cytogenetic testing may facilitate earlier intervention and improve long‐term outcomes. In parallel, multicenter collaborative studies are needed to better define the natural history of Swyer syndrome, identify predictors of malignant transformation, and determine whether specific genetic subtypes confer differential oncologic risk, thereby refining recommendations for surveillance, timing of gonadectomy, and individualized management.

Taken together, prompt recognition of hypergonadotropic hypogonadism, routine karyotype analysis in adolescents with primary amenorrhea, and timely prophylactic gonadectomy remain essential to improving endocrine and reproductive outcomes while preventing gonadal malignancy before invasive disease develops.

## Author Contributions

**Chukwuka Elendu:** conceptualization, investigation, data curation, methodology, formal analysis, writing – original draft, writing – review and editing, visualization, supervision, validation, project administration. **Alamjeet K. Sidhu:** investigation, validation, writing – original draft. **Olisa S. Okabekwa:** formal analysis, writing – review and editing, validation. **Ayi T. Debua:** investigation, validation, writing – review and editing. **Ahmed I. Ali:** supervision, writing – review and editing, validation.

## Funding

The authors have nothing to report.

## Disclosure

The views expressed in this paper are solely those of the authors and do not represent the official positions of any affiliated institutions. The authors declare no conflicts of interest and received no funding for this work.

## Ethics Statement

Ethics approval was not required, as this letter does not involve new patient data, human subject research, or original clinical investigation.

## Consent

Written informed consent for publication was obtained where applicable. This letter does not include new patient data.

## Data Availability

Data sharing not applicable to this article as no datasets were generated or analysed during the current study.
